# Healthcare and Social Needs of Older Adults in Underserved Urban Communities: Insights from Community Health Workers

**DOI:** 10.1007/s11524-025-01011-9

**Published:** 2025-09-27

**Authors:** Arkers Kwan Ching Wong, Tianyi Liu, Luna Ziqi Liu, Jonathan Bayuo, Xingjuan Tao, Frances Kam Yuet Wong

**Affiliations:** 1 School of Nursing, The Hong Kong Polytechnic University, 11 Cheong Wan Road, Hung Hom, Hong Kong; 2 School of Nursing, Shanghai Jiao Tong University, Shanghai, China

**Keywords:** Underserved urban areas, Economic disadvantaged older adults, Community health worker, Healthcare access, Social support, Healthcare delivery

## Abstract

As populations age globally, ensuring equitable healthcare access and social support for older adults in underserved urban areas has become increasingly critical. Elderly residents in low-income districts face challenges, including poor living conditions, social isolation, and healthcare access barriers. Community health workers (CHWs) are vital in bridging these gaps, yet their effectiveness is often Limited by resources and training. This qualitative study explores the healthcare and social needs of elderly residents receiving community services, identifies gaps in support systems, and examines the challenges faced by CHWs in delivering care in an underserved urban district. The study took place in Sham Shui Po, a district in Hong Kong with a high concentration of economically disadvantaged elderly residents. Data were collected through 17 semi-structured interviews with older residents and non-governmental organization (NGO) staff, alongside three focus group discussions with CHWs, and were analyzed using thematic analysis. The results showed that senior residents faced poor living conditions, chronic illnesses, and mobility issues, exacerbated by financial constraints and limited healthcare access. Long wait times, transportation challenges, and language barriers hindered medical service use. Many struggled with digital healthcare tools, limiting their ability to manage health independently. CHWs provided vital support but encountered physical strain, inadequate training, and logistical difficulties, highlighting the need for structured training and better resources. Addressing elderly care challenges requires integrated healthcare models, expanded financial and digital literacy programs, and enhanced CHW training and support. Strengthening these areas can improve health outcomes and well-being for aging populations in low-income urban settings.

## Introduction

Aging is a universal phenomenon that reflects advancements in healthcare and improved Living conditions, with the number of individuals aged 65 and older projected to reach 2.2 billion by 2050 [1]. This growing demographic presents opportunities to foster healthier, more active aging through proactive healthcare strategies and supportive communities. While older adults may face increased risks of chronic illnesses, functional decline, and multimorbidity, investing in preventive care, inclusive healthcare services, and social support can enhance their quality of life and overall well-being [2, 3]. As healthcare systems adapt to this demographic shift, ensuring equitable access to medical care and community-based resources will be key to empowering older adults to live independently and thrive [4, 5].

Hong Kong, one of the fastest-aging regions in the world, is projected to have over one-third of its population aged 65 and older by 2054 [6]. Despite its high level of economic development, significant socioeconomic inequalities persist, with approximately 45% of elderly residents living below the poverty line and relying heavily on public healthcare services and government assistance [7–9]. Compared to their more affluent counterparts, older adults facing economic hardship tend to have lower health literacy, that is, the ability to obtain, understand, and apply health-related information to make informed decisions about care. Limited health literacy has been associated with poor medication management, reduced engagement in preventive services, and greater difficulty navigating healthcare systems. These factors contribute to a higher prevalence of chronic conditions and reduced access to appropriate healthcare services among disadvantaged elderly populations [10]. Structural poverty, deeply embedded in the socioeconomic landscape, leaves many seniors with little choice but to live in substandard housing, consume less nutritious food, experience social isolation, and endure multiple physiological and psychological stressors—factors that further elevate their risk for poor health outcomes [11].

These issues, while examined here in Hong Kong, have implications that extend beyond local boundaries. Many rapidly aging societies—across East Asia, Europe, and North America—face parallel challenges, including widening health inequities, constrained healthcare access, and rising demand for community-based support. Hong Kong provides a valuable case because it combines advanced healthcare infrastructure with deeply entrenched socioeconomic disparities. Examining how underserved older adults and CHWs navigate these barriers yields transferable lessons for other urban settings worldwide.

The unequal distribution of healthcare resources further exacerbates these challenges, as wealthier individuals benefit from both public and private healthcare services, while those living in poverty often struggle to afford routine care and manage chronic conditions. Many rely on public healthcare services, which frequently face issues of accessibility and long waiting times [12, 13]. Among them, elderly populations in impoverished districts are particularly vulnerable due to barriers such as inadequate infrastructure, social isolation, and financial instability [14]. The absence of integrated healthcare and social support systems further limits access to essential services, creating a fragmented care network in which older adults face difficulties navigating healthcare services and obtaining necessary resources [15]. These inequalities in healthcare accessibility and affordability contribute to delayed treatment, increased disease burden, and a decline in overall well-being among elderly residents in underserved areas [16, 17]. Addressing these challenges requires community-driven approaches that consider the healthcare, social, and environmental needs of older adults, particularly those in marginalized and resource-limited communities.

Given these significant challenges, it is crucial to explore community health needs, which encompass not only the collective health challenges and requirements of specific populations but also the broader social, economic, and environmental factors that influence well-being [18]. Individuals within a particular community—whether defined by geography, shared interests, or social networks—often experience similar challenges due to common environmental and socioeconomic conditions. These challenges aggregate at the community level, reflecting the collective impact of individual hardships [18, 19]. Community health needs, therefore, extend beyond individual medical care to include determinants such as deprivation, housing conditions, and nutrition, all of which significantly shape overall health outcomes [18, 20]. While individual health needs have received considerable academic attention, addressing community health needs requires a more holistic approach—one that integrates multiple stakeholders, including residents, service providers, and community organizers. This approach considers the community’s conditions, resources, and environment as an interconnected whole, rather than focusing solely on individual members. However, despite its importance, this perspective is often overlooked, particularly when identifying needs and proposing actionable solutions tailored to specific communities.

### Study Objective and Research Questions

This study aims to explore community health needs in aged care within impoverished areas of Hong Kong, with a particular focus on districts with a high concentration of elderly residents. By integrating the perspectives of both elderly individuals and community service providers, the study seeks to develop a comprehensive understanding of the challenges faced by this population. Specifically, it examines the health needs of elderly residents receiving community services, identifies gaps and limitations in existing healthcare and social care services, and explores the barriers encountered in the care delivery process.

Guided by these objectives, the study addresses the following key research questions:

- What are the health needs of elderly individuals in the district?
- What gaps and limitations exist in the current healthcare and social care services?
- What barriers and challenges arise during the care delivery process, and what specific difficulties do CHWs encounter?

Through this exploration, the study aims to enhance integrated care by fostering collaboration and seamless transitions between healthcare and social services. By identifying community health needs, the findings will provide actionable insights to inform the development of more targeted, equitable, and sustainable service models. These insights will help decision-makers design healthcare systems that better support elderly residents in Hong Kong’s impoverished communities.

## Methods

### Study Design

This study employed a qualitative methodology to explore the community health needs of elderly residents in underserved districts of Hong Kong. To gather empirical data, two qualitative methods were utilized: individual interviews and focus group discussions.

Semi-structured individual interviews provided an opportunity for in-depth, one-on-one exchanges between the interviewer and participants. These interviews were guided by broad, open-ended questions designed to elicit deep insights into community health challenges. This method was chosen for its ability to capture detailed perspectives from community stakeholders with firsthand knowledge of local health issues.

In addition, focus group discussions were conducted to explore collective perceptions of community health needs. The interactive nature of focus groups facilitated dynamic discussions, allowing participants to share experiences and uncover social and communal factors influencing elderly health and well-being.

By integrating individual interviews and focus groups, the study aimed to present a comprehensive understanding of the challenges faced by elderly residents, CHWs, and service providers in delivering effective care. This multi-faceted approach ensured a well-rounded analysis of both individual experiences and broader community-level concerns. This study is reported according to the Standards for Reporting Qualitative Research (SRQR) checklist [21].

### Setting and Participants Recruitment

Participants were recruited from Sham Shui Po, a district in Hong Kong with a high concentration of economically disadvantaged older residents. According to the Census and Statistics Department (2022), 21.5% of the district’s population is aged 65 or older, with 45.6% of impoverished residents belonging to this age group [6]. To ensure a diverse representation of perspectives, convenience and purposive sampling strategies were employed to recruit participants from three key stakeholder groups: elderly individuals, CHWs, and community service providers from local NGOs.

### Data Collection

Data collection took place between June and December 2024, involving 37 participants: 15 elderly individuals, 20 CHWs, and 2 NGO service providers. Seventeen semi-structured interviews were conducted with elderly participants (*n* = 15) and service providers (*n* = 2), while the 20 CHWs participated exclusively in three focus group discussions. The groups were mutually exclusive, allowing for both in-depth individual narratives and interactive group dynamics, thereby supporting triangulation across individual, community, and organizational perspectives.

Three semi-structured interview guides were developed, sharing core domains such as health needs, healthcare access, and community resources, but tailored to each group’s unique role. For elderly participants, the guide emphasized daily living conditions, health challenges, and personal experiences of care. For CHWs, questions focused on caregiving roles, service delivery barriers, training needs, and coping strategies. For NGO service providers, the guide explored organizational challenges, interagency coordination, and unmet needs among elderly clients.

Interviews with elderly participants were conducted in their homes, in Cantonese or Mandarin, and lasted 25–60 min. Focus groups with CHWs took place in local community centers and lasted 60–90 min. All sessions were audio-recorded with participants’ consent to ensure accurate transcription and analysis.

### Data Analysis

The interviews and focus group discussions were digitally audio-recorded, transcribed verbatim in Cantonese, and de-identified to ensure participant anonymity. Two authors (TL and LZL) reviewed the transcripts for accuracy. After verification, the transcripts were translated into English by members of the research team, with both authors (AKCW and TL) independently reviewing the translations to ensure fidelity to the original meaning.

The de-identified, translated transcripts were then imported into NVivo 14 for thematic analysis. The analysis followed the framework outlined by Braun and Clarke (2006), involving the identification of codes, categories, sub-themes, and overarching themes [22]. Initial open coding was conducted by author TL, with validation and refinement performed by author AKCW. Any discrepancies in the analysis were resolved through discussion. NVivo 14 facilitated systematic data management, coding, and ensured rigor and consistency throughout the analysis process.

### Methodological Rigor/Trustworthiness

The trustworthiness of this study was evaluated based on four criteria: (1) credibility, (2) dependability, (3) confirmability, and (4) transferability [23]. To enhance credibility and dependability, summarized results were shared with participants for feedback and clarification. Audit trails and peer debriefings ensured consistency and confirmability during data analysis. Transferability was strengthened by providing detailed descriptions of the study’s context, methodology, and findings. The analysis was conducted in both Cantonese and English to maintain consistency, with fluent authors ensuring accurate interpretation. An iterative approach to analysis reinforced consistent coding, documented through an audit trail. A constant comparison approach extended the analysis beyond individual-level data to include comparisons within and across participant groups.

### Researcher Reflexivity

The research team comprised nursing and public health scholars with extensive experience in community-based health research and qualitative methodologies. While this expertise facilitated rapport with participants and supported nuanced interpretation of health-related issues, it also carried the potential for preconceptions to shape data collection and analysis. To enhance reflexivity, the researchers maintained reflexive journals during fieldwork, documenting assumptions, emotional responses, and possible biases. Regular peer debriefing sessions were conducted to critically examine how researcher perspectives might influence interpretation. In addition, the involvement of multiple analysts from different disciplinary backgrounds ensured that the coding and thematic development reflected diverse viewpoints rather than a single disciplinary lens.

### Ethical Considerations

The study was conducted in accordance with the principles of the Declaration of Helsinki and received ethical approval from the Ethics Committee of a university in Hong Kong (HSEARS20230825003-01). All eligible participants were informed of their right to decline participation and withdraw from the interview at any time. Written informed consent was obtained from all participants. To ensure privacy and confidentiality, all collected data were anonymized and securely stored, accessible only to members of the research team.

## Results

### Participant Demographic Data

A total of 37 participants were included: 15 elderly individuals, 20 CHWs, and 2 NGO service providers. The demographic breakdown reflects the study’s purposive sampling strategy to capture perspectives from both service recipients and providers. A relatively larger number of CHWs were recruited to allow for focus group discussions that could capture interactive dynamics and collective challenges in frontline care. Elderly participants were selected to represent variation in age, gender, marital status, and socioeconomic status, reflecting the diverse living circumstances of disadvantaged older residents in Sham Shui Po. Although only two service providers were interviewed, their inclusion provided organizational-level perspectives that complemented the lived experiences of elderly participants and CHWs. This balance of participants ensured triangulation across different stakeholder levels—individual, community, and organizational. Further details about the demographic characteristics of the participants are provided in Table 1.

**Table 1:** Demographic data of the participants

Characteristic	Elderly individuals	CHWs	Service providers
	(*n* = 15)	(*n* = 20)	(*n* = 2)
**Count/%**			
**Gender**			
Male	11 (73.3%)	1 (5.0%)	0
Female	4 (26.7%)	19 (95.0%)	2 (100%)
**Age, (years)**			
Mean (SD)	82.7 (7.69)	47.5 (9.29)	-
**Marital status**			
Single	2 (13.3%)	1 (5.0%)	-
Married	5 (33.3%)	17 (85.0%)	-
Divorced	0	1 (5.0%)	-
Widowed	8 (53.5%)	1 (5.0%)	-
**Education**			
No formal education	1 (6.7%)	0	-
Primary school	6 (40.0%)	1 (5.0%)	-
Middle school and high school	6 (40.0%)	13 (65.0%)	-
Tertiary education and above	2 (13.3%)	6 (30.0%)	-
**Financial status**			
More than sufficient	0	3 (15.0%)	-
Barely sufficient	13 (86.7%)	13 (65.0%)	-
Not sufficient	2 (13.3%)	3 (15.0%)	-
Extremely not sufficient	0	1 (5.0%)	-
**CHW experience**			
New entry	-	1 (5.0%)	-
6 months to 1 year	-	3 (15.0%)	-
1 to 2 years	-	4 (20.0%)	-
3 to 4 years	-	3 (15.0%)	-
More than 4 years	-	9 (45.0%)	-

### Qualitative Data Analysis

This study identified three primary themes and eight categories that provide valuable insights into elderly care and community services in underserved districts, addressing the three research questions. Table 2 reports the results of the thematic analysis. The themes are the following:

1. **Elderly population needs** — Examining the challenges posed by environmental conditions, social engagement, and healthcare access.
2. **Current healthcare services** — Highlighting barriers related to healthcare accessibility, financial constraints, and cultural and technological limitations.
3. **Caregiver challenges **— Exploring the difficulties faced by CHWs and their innovative approaches to addressing elderly needs.

**Table 2:** Thematic analysis results

Theme	Code
Elderly population needs	Environmental challenge and social engagement
	Healthcare needs
Barriers to current healthcare service barriers	Service accessibility
	Financial barriers
	Cultural and technological barriers
Caregiver challenges	The role of CHWs in bridging gaps
	Challenges in providing community service
	Training and support for CHWs

#### Theme 1: Elderly Population Needs

1. Environmental challenges and social engagement

Elderly participants described how financial hardship and poor housing conditions compromised their health and independence. Homeowners were especially disadvantaged, as they were often excluded from public housing support but still faced expensive maintenance costs. As one widowed participant explained:

*“I live alone, so managing my home is difficult, especially with repairs. Last time, it cost around ten thousand dollars, and I had to leave temporarily because I couldn’t afford to stay. Now, more repairs are needed, but it’s too expensive.” [FA013] *

CHWs also observed situations where elderly residents lacked even basic amenities, such as cooling equipment in summer, highlighting the severity of deprivation in some cases [CHW1-FG1].

In addition to environmental barriers, many elderly residents experienced weak social networks and inconsistent family support. While some valued the companionship provided by CHWs, they expressed a desire for longer and more regular visits. For example, one participant noted:

*“The community health workers are very kind, almost like friends… I just wish they could stay longer during their visits.” [FA001]*

Others reflected on diminished family ties, particularly when children had moved abroad:

*“My daughter lives abroad and rarely calls. The family bond is weak now; they have become very westernized.” [FA002]*

(2) Healthcare Needs

Multiple chronic conditions, mobility decline, and visual impairments were common among elderly participants, affecting both daily functioning and social engagement. Fear of falls emerged as a pervasive concern, with many participants describing inadequate home safety features. One elderly man recounted:

*“I slipped in the bathroom. The grab bars were too low, and I hit my head. I don’t take the emergency call button into the bathroom because there’s nowhere to hang it.” [FA002]*

Others emphasized the need for fall-prevention support and home modifications, with one participant suggesting:

*“It would be helpful to have someone teach me how to organize my home to prevent falls.” [FA011]*

#### Theme2: Barriers to Current Healthcare Service Barriers

(1) Service accessibility

Elderly residents frequently reported challenges in accessing healthcare due to long waiting times, travel difficulties, and rushed consultations. Such constraints left many feeling dissatisfied and underinformed about their conditions. As one participant explained:

*“Sometimes doctors don’t explain the test results. It’s frustrating not knowing my condition clearly.” [FA012]*

NGO staff also noted these concerns, observing that some elderly individuals were unaware of or reluctant to access available support, further limiting service utilization [Staff-1].

(2) Financial Barriers

Despite subsidies for those aged 75 and above, healthcare costs remained prohibitive for some, particularly in relation to medications. One participant reflected:

*“The doctor prescribed medication that costs about HKD 800 to 1000 a month. I had to buy it myself at first, even though it was necessary.” [FA001]*

(3) Cultural and Technological Barriers

Language barriers were particularly challenging for elderly residents who spoke dialects other than Cantonese, requiring CHWs to provide additional support [CHW1-FG2]. At the same time, the increasing reliance on digital healthcare platforms further excluded many elderly participants who lacked digital literacy. As one explained:

*“I don’t understand how to use these new systems. Sometimes the line is busy, and I don’t know what to do.” [FA001]*

#### Theme3: Caregiver Challenges

(1) The role of CHWs in Bridging Gaps

CHWs were widely recognized as a vital link between elderly residents and healthcare services, offering both emotional companionship and practical support. In some cases, their interventions were lifesaving, as when a CHW discovered an elderly man who had fallen at home and required urgent hospitalization [CHW1-FG3]. Many CHWs also described the personal fulfillment they derived from their work, despite its demands:

*“At first, I didn’t know how to care for the elderly, but I’ve learned a lot. I feel happy that I can support them while balancing my own family life.” [CHW1-FG2]*

(2) Challenges in Providing Community Service

The physical and emotional strain of caregiving was a recurrent theme, with CHWs reporting musculoskeletal pain from repetitive tasks. One participant noted:

*“We do the same activities every day, and my elbow is in pain.” [CHW4-FG1]*

NGO staff also highlighted high workloads and difficulties retaining staff, contributing to turnover and service instability [Staff-2].

(3) Training and Support for CHWs

CHWs consistently expressed a need for enhanced training, particularly in medical knowledge, medication safety, and psychological support skills. As one explained:

*“We don’t have enough medical knowledge. Sometimes an elderly person has multiple medications, and we’re unsure if they can be taken together.” [CHW4-FG2]*

They also emphasized the importance of communication training to build trust and provide reassurance to elderly residents [CHW5-FG2]. Access to basic equipment such as blood pressure monitors was identified as critical for improving care quality and early detection of health issues [CHW5-FG1].

## Discussion

This study examines the needs of elderly residents receiving community services, identifies gaps in existing healthcare and social support, and explores the barriers encountered in delivering care. Through an in-depth analysis of the social, environmental, and healthcare challenges faced by elderly individuals, as well as the obstacles service providers encounter, the findings highlight critical areas requiring urgent attention. The discussion is structured around three primary themes: (1) the social, environmental, and healthcare needs of the elderly population; (2) barriers to community health services, including accessibility, financial constraints, and cultural and technological challenges; and (3) the role and challenges of CHWs, particularly the necessity for their training and support.

### Elderly Population Needs: Social and Environmental Challenges

Elderly populations in underserved urban areas face substantial challenges related to their living environment and social isolation. These challenges are exacerbated by aging housing stock and deteriorating infrastructure, which often confine elderly residents within their homes and limit their access to essential healthcare services. The findings align with previous research demonstrating how poor housing conditions, such as the inability to afford necessary repairs, pose barriers to maintaining independence and health [24, 25]. Elderly homeowners, in particular, are disadvantaged, as they are ineligible for public housing assistance yet struggle with substandard living conditions that negatively impact their well-being.

Additionally, weakened family structures, due in part to younger generations’ migration and shifting caregiving responsibilities, have led to greater reliance on external care services. Many elderly individuals in the study reported infrequent contact with their children and limited family support, a phenomenon consistent with broader demographic trends [26]. This decline in family caregiving underscores the growing demand for professional in-home healthcare and comprehensive community support systems.

The study also highlights the increased risk of falls among elderly residents, a leading cause of injury and mortality in older adults. The Centers for Disease Control and Prevention (CDC) reported 38,742 fall-related deaths in the USA in 2021 (78.0 per 100,000 population) [27]. One in three community-dwelling adults aged 65 and older experiences at least one fall annually, with many going unreported due to fear or reluctance to seek medical attention [28, 29]. Given the significant burden of falls on healthcare systems and the elderly population, improving home safety and social support mechanisms is paramount in reducing fall incidence and associated health complications.

### Barriers to Community Healthcare Services

#### Healthcare Accessibility and Logistical Barriers

Older residents face considerable challenges in navigating the healthcare system, particularly those with mobility impairments or disabilities. Consistent with prior research emphasizing the need for improved healthcare accessibility and service efficiency in aging populations, participants expressed frustration with long waiting times, physical distances to healthcare facilities, and limited availability of nearby services, which discourage regular hospital or clinic visits [30]. These issues are compounded by understaffing and overcrowding in public healthcare settings, leading to delayed or inadequate medical care.

#### Financial Constraints

Despite government subsidies for individuals over 75, financial constraints remain a major barrier to healthcare access, particularly for those below the eligibility threshold. Many elderly individuals reported difficulties affording medications and treatments, leading some to forgo necessary care, exacerbating their health conditions. Previous research has similarly found that high out-of-pocket medical expenses deter elderly individuals from seeking timely healthcare services [31, 32]. This issue underscores the need for policy adjustments to expand financial assistance programs and ensure equitable healthcare access across age groups.

#### Cultural and Technological Barriers

Cultural and linguistic differences pose significant challenges for elderly residents, particularly those from immigrant backgrounds or with limited literacy in the dominant language. Language barriers hinder effective communication with healthcare providers, limiting patients’ understanding of their medical conditions and treatment options, and leading to reduced trust in the healthcare system [33].

Additionally, the increasing reliance on digital platforms for healthcare services further isolates older individuals unfamiliar with technology. Many participants reported difficulties navigating online appointment systems, digital payment platforms, and health applications, a finding supported by previous studies on digital health literacy challenges in aging populations [33]. These findings highlight the need for inclusive digital literacy programs tailored to elderly populations, enabling them to access essential healthcare resources more effectively.

### The Role and Challenges of CHWs

#### Bridging the Gap between Elderly Residents and Healthcare Services

CHWs play a critical role in addressing the healthcare and social needs of elderly individuals, providing both physical assistance and emotional support. The study’s findings demonstrate that CHWs foster strong relationships with elderly residents, helping them navigate complex healthcare systems while offering companionship and reassurance. Previous research has highlighted the significance of CHWs as primary points of contact for elderly individuals, providing comfort and stability in their daily lives [34, 35].

#### Challenges Faced by CHWs

Despite their essential role, CHWs encounter significant challenges, including physical strain from caregiving tasks and emotional exhaustion from supporting elderly individuals with chronic conditions. Many CHWs in the study reported experiencing musculoskeletal discomfort from repetitive tasks such as lifting and carrying [34]. Additionally, CHWs lack formal medical training, limiting their ability to provide comprehensive care in emergencies or complex health situations. Expanding CHW training in chronic disease management, medication adherence, and emergency response would improve their effectiveness in delivering high-quality care [35].

#### The Need for Training and Resource Allocation

CHWs require better access to medical knowledge, training, and essential equipment such as blood pressure monitors to enhance their service delivery. Many expressed a desire for professional development opportunities, including psychological training to help them support elderly residents experiencing loneliness, anxiety, or cognitive decline. Previous studies have emphasized that ongoing training and professional development are critical to improving CHWs’ job satisfaction, retention, and effectiveness in elderly care settings [34, 35].

### Implications of the Findings

The findings underscore the need for a more integrated and tailored approach to community-based healthcare for elderly populations. A collaborative care model, which links healthcare providers, social service agencies, and community organizations, would help address the diverse needs of elderly residents. Improving service accessibility, expanding financial support, and enhancing digital literacy are critical to reducing inequities and empowering older adults to manage their health more effectively.

Training and supporting CHWs should also be prioritized through structured programs that enhance medical knowledge, communication skills, and psychological support competencies. In practice, recommended curricula could include modules on chronic disease management (e.g., hypertension and diabetes), geriatric care, fall prevention, medication safety, and basic mental health support for anxiety, depression, and loneliness. Training should also address digital health literacy to enable CHWs to assist elderly residents in using health technologies and navigating online health systems. Evidence from other contexts suggests that modular training programs delivered over several weeks, combined with refresher workshops and ongoing supervision, can be effective in sustaining CHW performance and reducing burnout. Adapting such models to Hong Kong, while integrating locally relevant content and collaboration with NGOs and primary care providers, would provide a practical framework for strengthening CHW capacity.

These findings must also be interpreted within the broader political and organizational context of elderly care in Hong Kong. Public healthcare services remain heavily relied upon but are strained by high demand and long waiting times, while NGOs fill important service gaps through community-based programs. However, the overlap between public initiatives and NGO-led services often creates fragmented care pathways, leaving older adults and their families to navigate a complex and sometimes duplicative system. Better coordination between sectors—through shared referral systems, joint training, and integrated service delivery—would reduce inefficiencies and strengthen continuity of care for disadvantaged elderly residents. At the same time, such interorganizational cooperation is constrained by structural and bureaucratic boundaries, misaligned funding models, and differing organizational cultures. Public services operate under centralized policies with strict eligibility criteria, while NGOs often rely on short-term, project-based funding that restricts service sustainability and scope. These systemic barriers limit the depth and consistency of collaboration, underscoring the need for policy reforms and stable resource allocation to enable more effective and enduring partnerships. In addition, integrating qualitative insights with objective health measures could enrich understanding of elderly health in underserved communities. Such mixed-method approaches would allow policymakers and practitioners to triangulate subjective experiences with clinical evidence, thereby designing interventions that are both contextually appropriate and medically robust.

Beyond Hong Kong, the implications extend to other rapidly aging urban societies worldwide. Although these findings are rooted in the socio-economic conditions of Sham Shui Po, similar structural inequities and service gaps are evident in other disadvantaged urban areas. Future comparative research across multiple districts or cities would help distinguish which challenges are context-specific and which are universal, thereby informing the design of more adaptable community-based models of care. Moreover, structural poverty, fragmented health systems, and limited digital literacy are challenges shared by cities across East Asia, Europe, and North America. The reliance on CHWs to bridge gaps between disadvantaged elderly populations and healthcare systems is globally relevant, yet our findings emphasize that without adequate training, resources, and institutional support, their effectiveness remains constrained. Hong Kong’s unique combination of advanced healthcare infrastructure and entrenched social inequalities therefore provides transferable lessons for designing equitable, community-driven models of care in diverse international settings.

### Limitations

This study was conducted in a single urban district (Sham Shui Po), which may limit the applicability of the findings to other areas with different socio-cultural and healthcare contexts. While the rich qualitative data provide valuable insights into elderly care challenges in disadvantaged urban communities, future research should include comparative or multi-site studies across diverse districts and regions to explore the extent to which these findings are transferable. In addition, convenience and purposive sampling may have introduced selection bias, potentially excluding more isolated older individuals. Reliance on self-reported data may be subject to recall or social desirability bias, with participants possibly overestimating satisfaction or underreporting challenges. Furthermore, the study did not incorporate objective health measures (e.g., clinical data on chronic conditions, functional status, or fall records). The reliance on self-reported health information may limit the accuracy of some accounts. Future studies that integrate qualitative findings with clinical and functional measures would provide a more holistic understanding of elderly health and strengthen the evidence base for targeted community interventions.

## Conclusion

This study highlights the multifaceted challenges faced by elderly populations in underserved urban areas, emphasizing the urgent need for integrated, culturally sensitive, and accessible community-based health services. CHWs play a pivotal role in bridging service gaps; however, their effectiveness is hindered by inadequate training, limited resources, and physical strain. To enhance elderly care quality, targeted investments in CHW training, professional development, and resource allocation are essential. Strengthening community-based healthcare models will be critical in improving health outcomes and overall well-being among aging populations.

## Data Availability

Data are available upon reasonable request.
